# Author Correction: Hyperpolarised ^13^C-MRI identifies the emergence of a glycolytic cell population within intermediate-risk human prostate cancer

**DOI:** 10.1038/s41467-022-28979-1

**Published:** 2022-03-07

**Authors:** Nikita Sushentsev, Mary A. McLean, Anne Y. Warren, Arnold J. V. Benjamin, Cara Brodie, Amy Frary, Andrew B. Gill, Julia Jones, Joshua D. Kaggie, Benjamin W. Lamb, Matthew J. Locke, Jodi L. Miller, Ian G. Mills, Andrew N. Priest, Fraser J. L. Robb, Nimish Shah, Rolf F. Schulte, Martin J. Graves, Vincent J. Gnanapragasam, Kevin M. Brindle, Tristan Barrett, Ferdia A. Gallagher

**Affiliations:** 1 Department of Radiology, Addenbrooke’s Hospital and University of Cambridge, Cambridge, UK; 2 Cancer Research UK Cambridge Institute, University of Cambridge, Cambridge, UK; 3 Department of Pathology, Cambridge University Hospitals NHS Foundation Trust, Cambridge, UK; 4 Department of Urology, Cambridge University Hospitals NHS Foundation Trust, Cambridge, UK; 5 School of Allied Health, Anglia Ruskin University, Cambridge, UK; 6 Patrick G Johnston Centre for Cancer Research, Queen’s University Belfast, Belfast, UK; 7 Nuffield Department of Surgical Sciences, University of Oxford, John Radcliffe Hospital, Oxford, UK; 8 Centre for Cancer Biomarkers, University of Bergen, Bergen, Norway; 9 Department of Clinical Science, University of Bergen, Bergen, Norway; 10 GE Healthcare, Aurora, OH USA; 11 GE Healthcare, Munich, Germany; 12 Division of Urology, Department of Surgery, University of Cambridge, Cambridge, UK; 13 Cambridge Urology Translational Research and Clinical Trials Office, Cambridge Biomedical Campus, Addenbrooke’s Hospital, Cambridge, UK; 14 Department of Biochemistry, University of Cambridge, Cambridge, UK

**Keywords:** Cancer imaging, Cancer metabolism, Prostate cancer, Prostate, Cancer imaging, Cancer metabolism, Prostate cancer, Prostate

Correction to: *Nature Communications* 10.1038/s41467-022-28069-2, published online 24 January 2022.

In this article the author name Rolf F. Schulte was incorrectly written as Rolf S. Schulte. The original article has been corrected.

