# Supplementary material for: Hyperpolarised 13C-MRI identifies the emergence of a glycolytic cell population within intermediate-risk human prostate cancer
Source: Nat Commun. 2022 Jan 24;13:466. doi: 10.1038/s41467-022-28069-2 (PMC8786834; doi:10.1038/s41467-022-28069-2)
Supplement: Supplementary file 1 — Supplementary Information [file 41467_2022_28069_MOESM1_ESM.pdf]

Hyperpolarised ^{13}C-MRI identifies the emergence of a glycolytic cell population within intermediate-risk human prostate cancer

Nikita Sushentsev[1,2], Mary A. McLean[1,3], Anne Y. Warren[4], Arnold J. V. Benjamin[1], Cara Brodie[3], Amy Frary[1], Andrew B. Gill[1], Julia Jones[3], Joshua D. Kaggie[1], Benjamin W. Lamb[5,6], Matthew J. Locke[1], Jodi L. Miller[3], Ian G. Mills[7,8,9,10], Andrew N. Priest[1], Fraser J. L. Robb[11], Nimish Shah[4], Rolf S. Schulte[12], Martin J. Graves[1], Vincent J. Gnanapragasam[5,13,14], Kevin M. Brindle[3], Tristan Barrett[1*†], Ferdia A. Gallagher[1†]

[1] Department of Radiology, Addenbrooke's Hospital and University of Cambridge, Cambridge, UK

[2] World-Class Research Centre "Digital Biodesign and Personalized Healthcare", Sechenov First Moscow State Medical University, Moscow, Russian Federation

[3] Cancer Research UK Cambridge Institute, University of Cambridge, Cambridge, UK

[4] Department of Pathology, Cambridge University Hospitals NHS Foundation Trust, Cambridge, UK

[5] Department of Urology, Cambridge University Hospitals NHS Foundation Trust, Cambridge, UK

[6] School of Allied Health, Anglia Ruskin University, Cambridge, UK

[7] Patrick G Johnston Centre for Cancer Research, Queen's University Belfast, Belfast, UK

[8] Nuffield Department of Surgical Sciences, University of Oxford, John Radcliffe Hospital, Oxford, UK

[9] Centre for Cancer Biomarkers, University of Bergen, Bergen, Norway

[10] Department of Clinical Science, University of Bergen, Bergen, Norway

[11] GE Healthcare, Aurora, OH, USA

[12] GE Healthcare, Munich, Germany

[13] Division of Urology, Department of Surgery, University of Cambridge, Cambridge, UK

[14] Cambridge Urology Translational Research and Clinical Trials Office, Cambridge Biomedical Campus, Addenbrooke's Hospital, Cambridge, UK

Corresponding author: Dr Tristan Barrett (tb507@medschl.cam.ac.uk)

[†]These authors contributed equally to this work

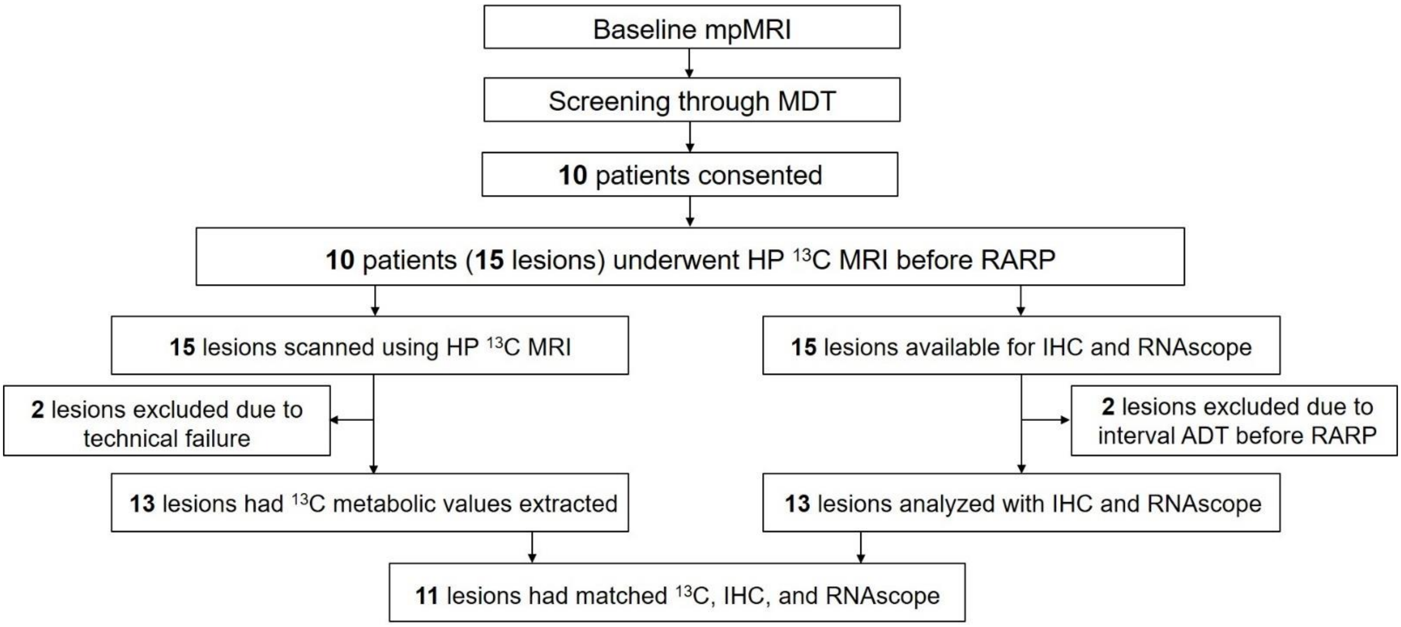

Supplementary Figure 1. Study flow chart.

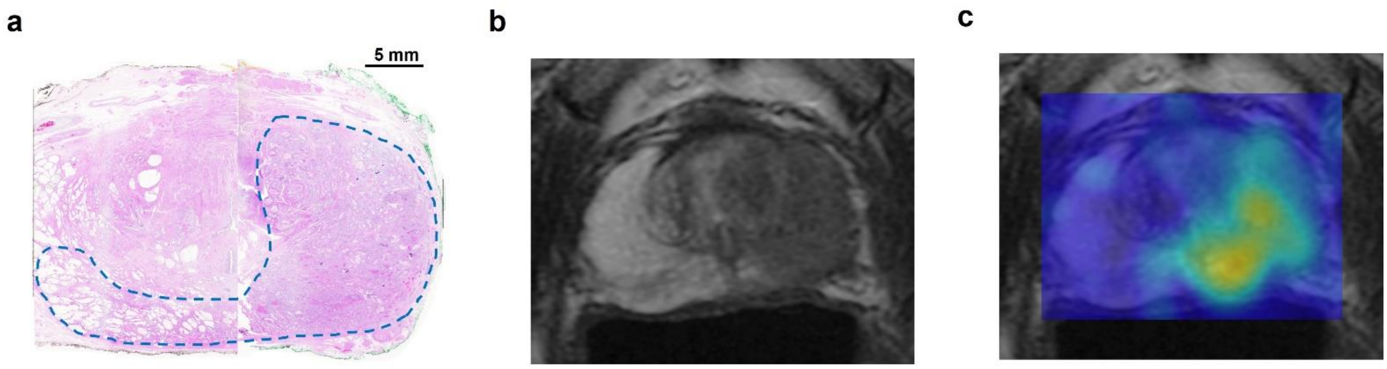

Supplementary Figure 2. Lesion excluded from the final HP ^{13}C-MRI analysis due to poor reliability of the underlying metabolic data. (a) A 67-year-old patient underwent robot-assisted radical prostatectomy with post-surgical pathologic assessment confirming the diagnosis of a grade group 5 adenocarcinoma of the prostate involving the whole left side of the gland and extending into the right peripheral zone (blue ROI; tumour 8 in Table S1). The image is comprised of two separate haematoxylin-and-eosin slides cut from prostatectomy formalin-fixed, paraffin-embedded blocks used for IHC and RNAscope analysis. **(b)** T_2-weighted imaging demonstrating a large Prostate Imaging-Reporting and Data System (PI-RADS) 5 lesion located in the left mid/apex peripheral zone. **(c)** Total carbon SNR map demonstrating an area of high signal corresponding to the tumour location on the pathology specimen and T_2-weighted imaging. The mean total carbon SNR value derived from the tumour ROI was 3.2, which is below the acceptable threshold of 5.0, which prompted us to exclude this case from HP ^{13}C-MRI analysis.

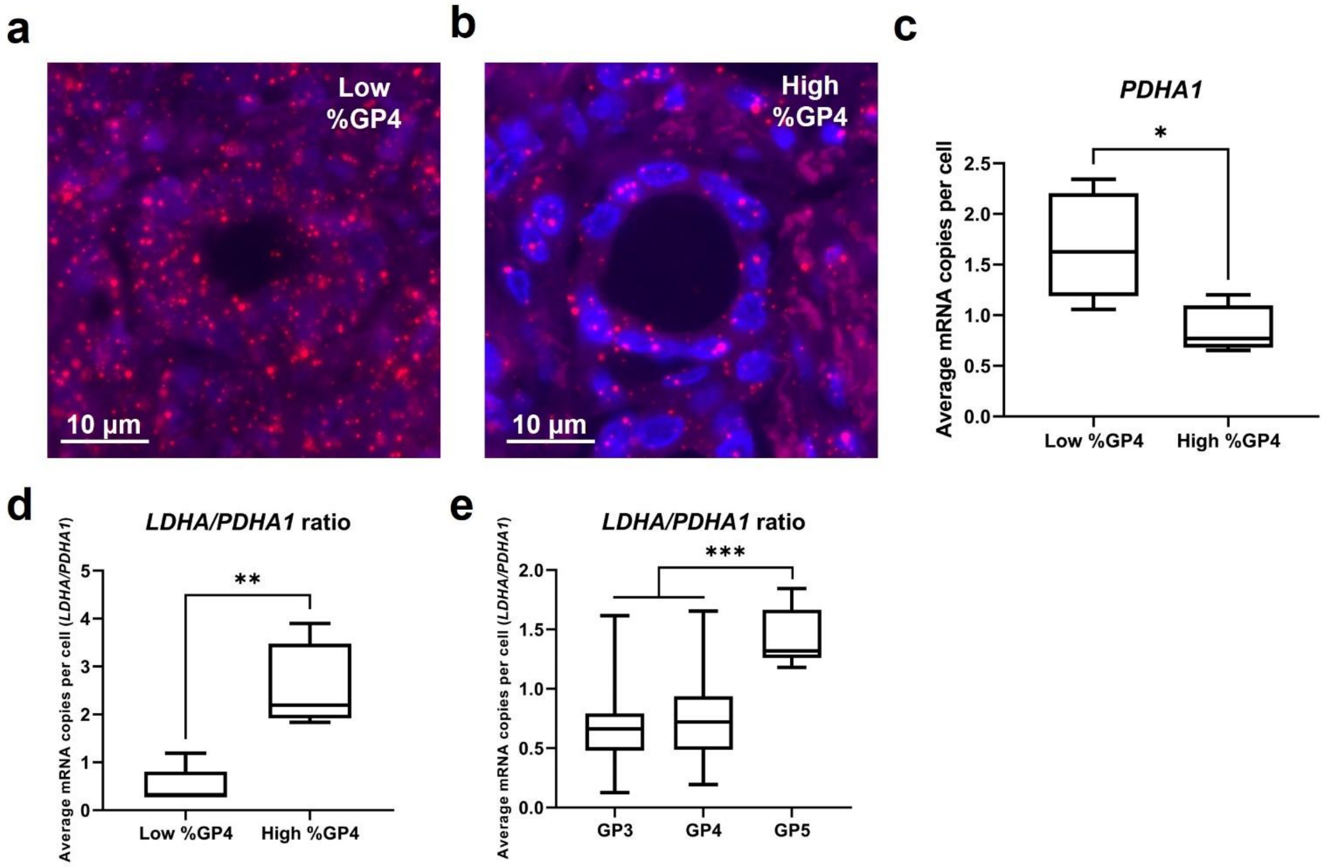

Supplementary Figure 3. Comparison of *PDHA1* mRNA expression and *LDHA*/*PDHA1* ratio in tumours with low and high percentage Gleason pattern 4, as well as malignant glands consisting of Gleason pattern 3, 4, and 5 disease. (a, b) Representative images of immunofluorescent RNAscope *PDHA1* staining (red) in malignant glands obtained from low %GP4 (tumour 2; **Supplementary Table 2**) and high %GP4 (tumour 4; **Supplementary Table 2**) lesions, respectively. While the images were obtained from ROIs encompassing GP3 and GP4 disease, respectively, they are not suitable for diagnostic grading due to high magnification used to showcase specific mRNA expression patterns and the presence of *PDHA1* and DAPI staining only. **(c)** RNAscope-derived mRNA *PDHA1* expression, measured as the average mRNA copies per cell, in tumours with low (≤10%) and high (>10%) %GP4. Data in panels **(c-d)** were derived from n = 10 biologically independent samples. **(d)** *LDHA*/*PDHA1* ratio between tumours with low and high %GP4. **(e)** *LDHA*/*PDHA1* ratio in Gleason pattern 3, 4, and 5 glands, representing n = 67 biologically independent samples. Centre lines represent the median values, boxes denote the interquartile ranges (25th to 75th percentiles), and whiskers denote the minimum and maximum values. * P = 0.012; ** P = 0.006, and *** P = 0.0004 were derived using the two-sided Mann-Whitney U test. Source data are provided as a **Source Data** file.

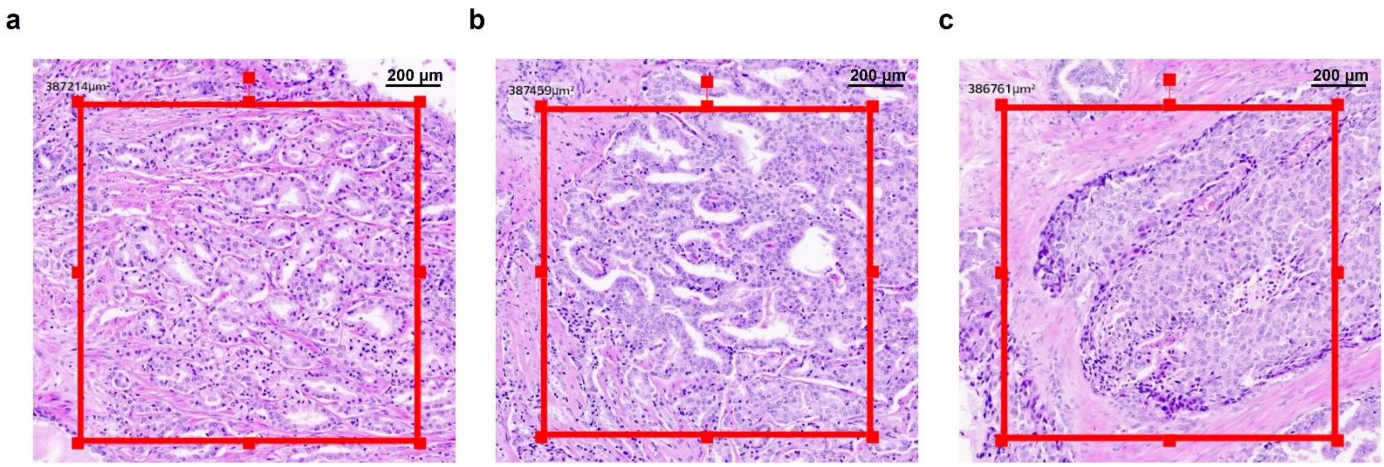

Supplementary Figure 4. Representative examples of areas harboring Gleason pattern 3, 4, and 5 glands used for IHC and RNAscope analyses. Representative H&E image showcasing sample areas consisting of: **(a)** GP3, **(b)** GP4, and **(c)** GP5 glands used for IHC and RNAscope analyses detailed in the main text.

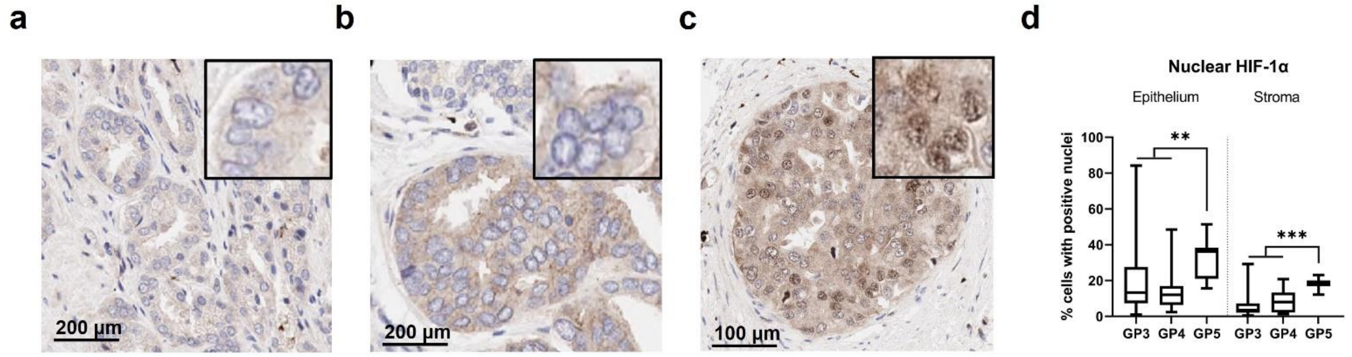

Supplementary Figure 5. Comparison of the nuclear HIF-1α expression in malignant glands consisting of Gleason pattern 3, 4, and 5 disease. (a-c) Representative images of IHC nuclear HIF-1α staining in GP3, GP4, and GP5 glands, respectively. The black boxes include highly magnified images of individual nuclei, demonstrating a prominent increase in nuclear HIF-1α staining in GP5 disease compared to both GP3 and GP4 glands. **(d)** Cell-specific IHC-derived expression of the nuclear fraction of HIF-1α, measured as percentage cells with positive nuclei, in GP3, GP4, and GP5 glands, representing n = 68 biologically independent samples. Centre lines represent the median values, boxes denote the interquartile ranges (25th to 75th percentiles), and whiskers denote the minimum and maximum values. ** $P = 0.001$ and *** $P = 0.0002$ were derived using the two-sided Mann-Whitney U test. Source data are provided as a **Source Data** file.

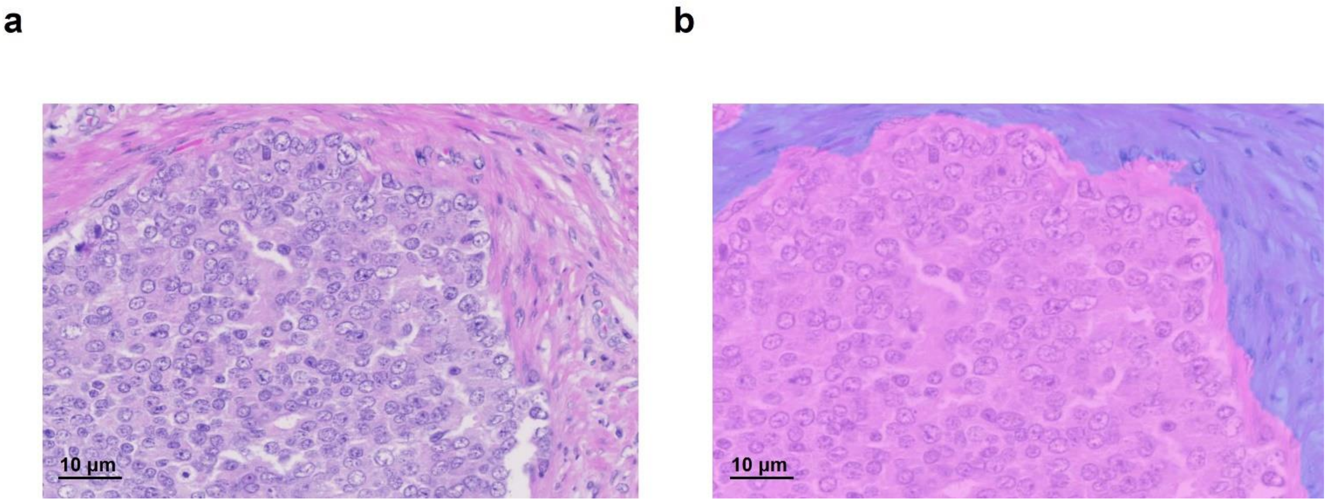

Supplementary Figure 6. Representative examples of epithelial and stromal cell differentiation using a random forest classifier. (a) Representative haematoxylin-and-eosin image of a tumour focus containing a GP5 gland with **(b)** a superimposed random forest classifier demonstrating automated detection of epithelial (pink) and stromal (blue) cell populations. The same classifier was applied to IHC and RNAscope images[1].

Patient	Tumour	PSA, ng/ml	PZ/TZ	Final ISUP grade group	%GP4	Tumour volume, mm^3	Polarisation, %	Mean total carbon SNR
1	1	8.5	PZ	2	<5	270	21	49
	2		PZ	2	<5	653		53
2	3	6.6	PZ	2	10	486	20	87
	4		PZ	3	60	3846		89
3	5	4.7	PZ	2	5	446	19	39
	6		TZ	1	0	472		27
4	7	13.9	TZ	2	15	1363	23	37
5	8	19.1	TZ	2	30	2789	28	51
	9		PZ	2	20	228		77
6	10	12.5	TZ	2	<5	5229	28	112
7	11	7.6	PZ	2	<5	1492	7	29
8	12	3.1	PZ	3	50	2179	33.8	62
	13		TZ	1	0	705		54
9	14[†]	6.9	PZ	5	-	6410	16	4
10	15[†]	6.6	PZ	5	-	7771	28	3

Supplementary Table 1. Summary characteristics of the study cohort. [†] denotes lesions excluded from the HP ^{13}C-MRI image analysis due to the technical failure; these lesions are, therefore, not listed in **Table 1** of the main text. PZ: peripheral zone. TZ: transition zone. PSA: prostate-specific antigen. SNR: signal-to-noise ratio.

Correlation pair	Spearman's ρ	95% confidence interval of Spearman's ρ	*P*
Lactate SNR and pyruvate SNR	0.43	-0.21 to 0.81	0.17
Lactate SNR and total carbon SNR	0.64	0.08 to 0.89	0.03
Lactate SNR and k_{PL}	0.25	-0.39 to 0.73	0.43
Lactate SNR and mean ADC	-0.69	-0.91 to -0.17	0.02
Lactate SNR and %GP4	0.65	0.11 to 0.90	0.03
Pyruvate SNR and total carbon SNR	0.90	0.67 to 0.97	<0.0001
Pyruvate SNR and k_{PL}	0.53	-0.08 to 0.85	0.08
Pyruvate SNR and mean ADC	0.01	-0.58 to 0.59	0.99
Pyruvate SNR and %GP4	0.38	-0.26 to 0.79	0.22
Total carbon SNR and k_{PL}	0.46	0.05 to 0.88	0.13
Total carbon SNR and mean ADC	-0.22	-0.17 to 0.82	0.49
Total carbon SNR and %GP4	0.40	-0.25 to 0.80	0.20
k_{PL} and mean ADC	-0.14	-0.67 to 0.49	0.67
k_{PL} and %GP4	0	-0.59 to 0.59	1
Mean ADC and %GP4	-0.62	-0.89 to -0.06	0.03

Supplementary Table 2. Outputs of the Spearman's correlation analysis underlying **Figure 2a** of the main text.

**Correlation pair**	**Spearman's ρ**	**95% confidence interval of Spearman's ρ**	***P***
Lactate SNR and pyruvate SNR	0.43	-0.21 to 0.81	0.17
Lactate SNR and total carbon SNR	0.64	0.08 to 0.89	0.03
Lactate SNR and k_{PL}	0.25	-0.39 to 0.73	0.43
Lactate SNR and tumour epithelial cell number	0.78	0.42 to 0.94	0.002
Lactate SNR and tumour stromal cell number	0.52	-0.06 to 0.84	0.07
Lactate SNR and tumour epithelial/stromal cell ratio	0.36	-0.25 to 0.77	0.22
Lactate SNR and epithelial *LDHA*	0.52	-0.14 to 0.86	0.11
Lactate SNR and epithelial *LDHB*	-0.43	-0.82 to 0.25	0.19
Lactate SNR and combined epithelial LDH	0.99	0.96 to 0.10	< 0.0001
Lactate SNR and epithelial MCT1	-0.15	-0.69 to 0.51	0.67
Lactate SNR and stromal MCT4	0.01	-0.61 to 0.62	0.99
Lactate SNR and epithelium-to-stroma MCT4 ratio	0.90	-	0.002
Pyruvate SNR and k_{PL}	0.53	-0.08 to 0.85	0.08
Pyruvate SNR and tumour epithelial cell number	0.26	-0.36 to 0.72	0.39
Pyruvate SNR and tumour stromal cell number	0.03	-0.55 to 0.58	0.94
Pyruvate SNR and tumour epithelial/stromal cell ratio	0.25	-0.36 to 0.72	0.40
Pyruvate SNR and epithelial *LDHA*	0.64	0.04 to 0.90	0.04
Pyruvate SNR and epithelial *LDHB*	-0.07	-0.66 to 0.57	0.84
Pyruvate SNR and combined epithelial LDH	0.59	-0.04 to 0.88	0.06
Pyruvate SNR and epithelial MCT1	-0.02	-0.62 to 0.60	0.97
Pyruvate SNR and stromal MCT4	-0.02	-0.62 to 0.60	0.97
Pyruvate SNR and epithelium-to-stroma MCT4 ratio	0.67	-	0.06
k_{PL} and tumour epithelial cell number	0.25	-0.36 to 0.72	0.40
k_{PL} and tumour stromal cell number	0.30	-0.32 to 0.74	0.33
k_{PL} and tumour epithelial/stromal cell ratio	-0.23	-0.70 to 0.38	0.45
k_{PL} and epithelial *LDHA*	0.66	0.07 to 0.91	0.03
k_{PL} and epithelial *LDHB*	-0.08	-0.66 to 0.56	0.82
k_{PL} and combined epithelial LDH	0.14	-0.52 to 0.69	0.69
k_{PL} and epithelial MCT1	-0.56	-0.87 to 0.08	0.08
k_{PL} and stromal MCT4	-0.76	-0.94 to -0.28	0.009
k_{PL} and epithelium-to-stroma MCT4 ratio	-0.15	-	0.71
Tumour epithelial cell number and tumour stromal cell number	0.66	0.15 to 0.89	0.02
Tumour epithelial cell number and tumour epithelial/stromal cell ratio	0.39	-0.23 to 0.78	0.20
Tumour epithelial cell number and epithelial *LDHA*	0.46	-0.21 to 0.84	0.16
Tumour epithelial cell number and epithelial *LDHB*	-0.23	-0.74 to 0.45	0.50
Tumour epithelial cell number and combined epithelial LDH	0.88	0.59 to 0.97	0.001
Tumour epithelial cell number and epithelial MCT1	-0.06	-0.65 to 0.58	0.88
Tumour epithelial cell number and stromal MCT4	-0.26	-0.75 to 0.42	0.45

Tumour epithelial cell number and epithelium-to-stroma MCT4 ratio	0.92	-	0.001
Tumour stromal cell number and tumour epithelial/stromal cell ratio	-0.36	-0.77 to 0.26	0.23
Tumour stromal cell number and epithelial *LDHA*	0.35	-0.34 to 0.79	0.30
Tumour stromal cell number and epithelial *LDHB*	-0.03	-0.63 to 0.60	0.95
Tumour stromal cell number and combined epithelial LDH	0.43	-0.25 to 0.82	0.19
Tumour stromal cell number and epithelial MCT1	0.15	-0.51 to 0.70	0.67
Tumour stromal cell number and stromal MCT4	-0.55	-0.87 to 0.10	0.09
Tumour stromal cell number and epithelium-to-stroma MCT4 ratio	0.55	-	0.13
Tumour epithelial/stromal cell ratio and epithelial *LDHA*	0.06	-0.57 to 0.65	0.86
Tumour epithelial/stromal cell ratio and epithelial *LDHB*	-0.34	-0.79 to 0.35	0.31
Tumour epithelial/stromal cell ratio and combined epithelial LDH	0.51	-0.15 to 0.86	0.11
Tumour epithelial/stromal cell ratio and epithelial MCT1	-0.12	-0.68 to 0.53	0.74
Tumour epithelial/stromal cell ratio and stromal MCT4	0.33	-0.36 to 0.78	0.33
Tumour epithelial/stromal cell ratio and epithelium-to-stroma MCT4 ratio	0.63	-	0.08
Epithelial *LDHA* and epithelial *LDHB*	0.10	-0.55 to 0.67	0.78
Epithelial *LDHA* and combined epithelial LDH	0.54	-0.11 to 0.87	0.09
Epithelial *LDHA* and epithelial MCT1	-0.47	-0.85 to 0.20	0.15
Epithelial *LDHA* and stromal MCT4	-0.57	-0.88 to 0.06	0.07
Epithelial *LDHA* and epithelium-to-stroma MCT4 ratio	0.30	-	0.44
Epithelial *LDHB* and combined epithelial LDH	-0.41	-0.82 to 0.27	0.21
Epithelial *LDHB* and epithelial MCT1	0.01	-0.61 to 0.62	0.99
Epithelial *LDHB* and stromal MCT4	-0.18	-0.72 to 0.49	0.56
Epithelial *LDHB* and epithelium-to-stroma MCT4 ratio	-0.43	-	0.25
Combined epithelial LDH and epithelial MCT1	-0.11	-0.68 to 0.54	0.76
Combined epithelial LDH and stromal MCT4	-0.01	-0.62 to 0.61	0.99
Combined epithelial LDH and epithelium-to-stroma MCT4 ratio	0.93	-	0.001
Epithelial MCT1 and stromal MCT4	0.46	-0.22 to 0.835	0.16
Epithelial MCT1 and epithelium-to-stroma MCT4 ratio	0.48	-	0.19
Stromal MCT4 and epithelium-to-stroma MCT4 ratio	0.13	-	0.74

Supplementary Table 3. Outputs of the Spearman's correlation analysis underlying **Figure 2b** of the main text.

**Correlation pair**	**Spearman's ρ**	**95% confidence interval of Spearman's ρ**	***P***
Lactate SNR and K^{trans}	-0.01	-0.62 to 0.61	0.99
Pyruvate SNR and K^{trans}	-0.14	-0.69 to 0.52	0.69
Total carbon SNR and K^{trans}	-0.21	-0.71 to 0.39	0.72
k_{PL} and K^{trans}	-0.23	-0.75 to 0.43	0.45

Supplementary Table 4. Outputs of the Spearman's correlation analysis between HP ^{13}C-MRI metabolic parameters and K^{trans}.

**Overall % positive cells**		***P***	**MCT1, % positive cells**		***P***	**MCT4, % positive cells**		***P***
MCT1	MCT4		Epithelium	Stroma		Epithelium	Stroma	
19.83 (9.67-44.78)	10.04 (4.79-26.24)	0.08	47.33 (14.05-62.38)	1.31 (0.64-5.64)	<0.0001	1.84 (1.33-10.50)	26.19 (20.89-42.93)	<0.0001

**Overall average mRNA copies per cell**		***P***	***LDHA*, average mRNA copies per cell**		***P***	***LDHB*, average mRNA copies per cell**		***P***
LDHA	*LDHB*		Epithelium	Stroma		Epithelium	Stroma	
0.72 (0.51-1.56)	1.92 (0.99-3.40)	0.01	0.85 (0.41-1.57)	0.82 (0.62-1.64)	0.55	2.10 (0.83-3.52)	1.76 (1.03-3.29)	0.84

**Overall MCT1, % positive cells**		***P***	**Epithelial MCT1, % positive cells**		***P***	**Stromal MCT1, % positive cells**		***P***
Low %GP4	High %GP4		Low %GP4	High %GP4		Low %GP4	High %GP4	
11.66 (6.48-32.91)	44.21 (23.31-49.64)	0.17	15.90 (8.97-68.26)	58.81 (42.17-70.72)	0.35	0.64 (0.10-2.95)	4.84 (1.59-5.64)	0.26

**Overall MCT4, % positive cells**		***P***	**Epithelial MCT4, % positive cells**		***P***	**Stromal MCT4, % positive cells**		***P***
Low %GP4	High %GP4		Low %GP4	High %GP4		Low %GP4	High %GP4	
4.93 (4.29-9.55)	32.12 (14.68-41.87)	0.02	1.33 (1.21-1.47)	13.23 (9.29-18.00)	0.01	23.47 (20.04-27.86)	46.78 (27.03-49.12)	0.17

**Epithelial *LDHA*, average mRNA copies per cell**		***P***	**Epithelial *LDHB*, average mRNA copies per cell**		***P***	**Epithelial *LDHA/LDHB ratio***		***P***
Low %GP4	High %GP4		Low %GP4	High %GP4		Low %GP4	High %GP4	
0.67 (0.56-0.87)	1.93 (1.50-2.83)	0.01	2.65 (2.08-3.08)	0.77 (0.71-0.82)	0.04	0.31 (0.20-0.56)	2.60 (1.16-3.26)	0.01

**Epithelium-to-stroma MCT4 ratio**		***P***	**Combined epithelial LDH, 10^3 mRNA copies**		***P***
Low %GP4	High %GP4		Low %GP4	High %GP4	
0.05 (0.05-0.07)	0.37 (0.25-0.42)	0.01	70.92 (55.85-90.66)	177.1 (148.6-206.8)	0.006

Supplementary Table 5. Analysis of the overall and compartmentalised expression patterns of MCT1, MCT4, epithelial *LDHA,* epithelial *LDHB*, and combined epithelial LDH, alongside their intergroup comparison between tumours with high and low percent Gleason pattern 4. The data are presented as median (interquartile range). *P* values were calculated using the two-sided Mann-Whitney U test. The table supports **Figure 3** of the main text.

PDHA1, average mRNA copies per cell		
**Low %GP4**	**High %GP4**	***P***
1.63 (1.19-2.21)	0.77 (0.69-1.10)	0.019
***LDHA/PDHA1* ratio**		
**Low %GP4**	**High %GP4**	***P***
0.31 (0.29-0.81)	2.20 (1.92-3.48)	0.006

Supplementary Table 6. Comparison of RNAscope-derived *PDHA1* mRNA expression in tumours with low and high percent Gleason pattern 4 (supporting Supplementary Figure 3). The data are presented as median (interquartile range). *P* values were calculated using the two-sided Mann-Whitney U test.

GP3 glands	GP4 glands	GP5 glands	*P* GP3 vs. GP4	*P* GP4 vs. GP5	*P* GP3 vs. GP5
**Overall MCT1, % positive cells**					
32.57 (5.48-54.46)	37.49 (13.10-48.81)	53.53 (46.88-58.06)	0.19	0.05	0.08
**Epithelial MCT1, % positive cells**					
48.79 (10.24-75.04)	41.02 (17.27-48.24)	67.33 (62.15-82.57)	0.64	0.02	0.08
**Stromal MCT1, % positive cells**					
1.29 (0.16-4.88)	2.50 (0.76-6.30)	10.64 (8.52-12.76)	0.09	0.04	0.003
**Overall MCT4, % positive cells**					
9.03 (5.89-18.32)	7.72 (4.60-19.74)	79.55 (63.81-81.40)	0.61	<0.0001	<0.0001
**Epithelial MCT4, % positive cells**					
2.49 (1.10-6.22)	2.31 (1.43-9.69)	38.30 (14.99-48.54)	0.63	0.002	0.0003
**Stromal MCT4, % positive cells**					
22.79 (18.96-37.44)	31.99 (22.03-38.49)	80.61 (67.98-83.00)	0.22	<0.0001	<0.0001
**Epithelial *LDHA*, average mRNA copies per cell**					
0.69 (0.34-1.03)	0.72 (0.56-0.99)	2.72 (2.29-2.94)	0.28	0.0006	<0.0001
**Epithelial *LDHB*, average mRNA copies per cell**					
1.74 (0.88-2.43)	1.35 (0.86-2.19)	1.04 (0.68-1.25)	0.48	0.05	0.01
**Combined epithelial LDH, 10^3 mRNA copies**					
4.12 (2.37-7.29)	6.85 (3.12-14.01)	123.8 (119.3-132.6)	0.07	<0.0001	<0.0001
**Epithelial *LDHA*/*LDHB* ratio**					
0.44 (0.28-0.70)	0.55 (0.36-0.75)	2.44 (1.94-3.14)	0.18	0.0003	<0.0001
**Epithelium-to-stroma MCT4 ratio**					
0.13 (0.05-0.21)	0.12 (0.05-0.25)	0.48 (0.22-0.58)	0.85	0.02	0.004

Supplementary Table 7. Comparison of MCT1, MCT4, *LDHA*, and *LDHB* expression patterns in malignant glands harboring Gleason pattern 3, 4, and 5 disease (supporting Figure 4 of the main text). The data are presented as median (interquartile range). *P* values were calculated using the two-sided Mann-Whitney U test.

GP3 glands	GP4 glands	GP5 glands	***P*** **GP3 vs. GP4**	***P*** **GP4 vs. GP5**	***P*** **GP3 vs. GP5**
**Epithelial HIF-1α, % cells with positive nuclei**					
13.25 (7.39-27.55)	12.03 (6.35-16.96)	36.33 (21.08-38.46)	0.48	0.001	0.006
**Stromal HIF-1α, % cells with positive nuclei**					
3.47 (2.02-7.27)	8.04 (2.17-13.30)	17.83 (16.85-20.02)	0.066	0.0002	0.0003

Supplementary Table 8. Comparison of the nuclear HIF-1$^{\alpha}$ immunohistochemical expression in malignant glands harboring Gleason pattern 3, 4, and 5 disease (supporting Supplementary Figure 5). The data are presented as median (interquartile range). *P* values were calculated using the two-sided Mann-Whitney U test.

***LDHA/PDHA1* ratio**					
**GP3 glands**	**GP4 glands**	**GP5 glands**	***P***<br>**GP3 vs. GP4**	***P***<br>**GP4 vs. GP5**	***P***<br>**GP3 vs. GP5**
0.66<br>(0.48-0.79)	0.72<br>(0.49-0.94)	1.32<br>(1.26-1.67)	0.35	0.0004	<0.0001

Supplementary Table 9. Comparison of RNAscope-derived LDHA/*PDHA1* ratios in malignant glands harboring Gleason pattern 3, 4, and 5 disease (supporting Supplementary Figure 3). The data are presented as median (interquartile range). *P* values were calculated using the two-sided Mann-Whitney U test.

Primary GP3 tumours (n = 197)	Primary GP4 tumours (n = 250)	Primary GP5 tumours (n = 50)	*P* GP3 vs. GP4	*P* GP3 vs. GP5	*P* GP4 vs. GP5
***SLC16A1* (MCT1)**					
1142.0 (774.2-1774.0)	1087.0 (732.3-1847.0)	1482.0 (678.5-2487.0)	0.60	0.20	0.16
1448.0±1070.0	1452.0±1081.0	1870.0±1679.0			
***SLC16A3* (MCT4)**					
175.7 (117.7-283.2)	200.7 (141.2-318.4)	300.2 (185.3-431.1)	0.021	<0.0001	0.0008
224.2±164.9	254.4±177.4	364.0±269.2			
LDHA					
5926.0 (5021.0-7181.0)	6539.0 (4898.0-8133.0)	6474.0 (4998.0-9694.0)	0.040	0.07	0.32
6300.0±2064.0	7009.0±3239.0	8685.0±7435.0			
LDHB					
3534.0 (2259.0-5118.0)	2342.0 (1368.0-3942.0)	2871.0 (1077.0-4115.0)	<0.0001	0.005	0.94
3746.0±2043.0	2884.0±1986.0	2870.0±1980.0			

Supplementary Table 10. Analysis of the TCGA RNA sequencing profiles of *SLC16A1*, *SLC16A3*, *LDHA*, and *LDHB* derived from prostatectomy samples of patients with prostate cancer (supporting Figure 6 of the main text). The data are presented as median (interquartile range) and mean ± standard deviation. *P* for comparisons between the median values were calculated using the two-sided Mann-Whitney U test. The raw data behind this table were originally obtained as part of the TCGA-PRAD study and downloaded from the CBioPortal (see references 57, 58 in the main text). The data from one lesion were excluded from the presented analysis because of the primary Gleason pattern being 2, which is not recommended for clinical use[2].

Supplementary References:

1. Brodie, C. Overcoming Autofluorescence (AF) and Tissue Variation in Image Analysis of In Situ Hybridization. *Methods Mol. Biol.* **2148**, 19–32 (2020).
2. Gordetsky, J. & Epstein, J. Grading of prostatic adenocarcinoma: current state and prognostic implications. *Diagn. Pathol.* **11**, (2016).
