# Supplementary material for: Hyperpolarised 13C-MRI identifies the emergence of a glycolytic cell population within intermediate-risk human prostate cancer
Source: Nat Commun. 2022 Jan 24;13:466. doi: 10.1038/s41467-022-28069-2 (PMC8786834; doi:10.1038/s41467-022-28069-2)
Supplement: Supplementary file 3 — Reporting Summary [file 41467_2022_28069_MOESM3_ESM.pdf]

nature portfolio

Corresponding author(s): Dr Tristan Barrett

Last updated by author(s): Nov 5, 2021

Reporting Summary

Nature Portfolio wishes to improve the reproducibility of the work that we publish. This form provides structure for consistency and transparency in reporting. For further information on Nature Portfolio policies, see our Editorial Policies and the Editorial Policy Checklist.

Statistics

For all statistical analyses, confirm that the following items are present in the figure legend, table legend, main text, or Methods section.

n/a	Confirmed	
☐	☒	The exact sample size (*n*) for each experimental group/condition, given as a discrete number and unit of measurement
☐	☒	A statement on whether measurements were taken from distinct samples or whether the same sample was measured repeatedly
☐	☒	The statistical test(s) used AND whether they are one- or two-sided<br>*Only common tests should be described solely by name; describe more complex techniques in the Methods section.*
☒	☐	A description of all covariates tested
☐	☒	A description of any assumptions or corrections, such as tests of normality and adjustment for multiple comparisons
☐	☒	A full description of the statistical parameters including central tendency (e.g. means) or other basic estimates (e.g. regression coefficient) AND variation (e.g. standard deviation) or associated estimates of uncertainty (e.g. confidence intervals)
☐	☒	For null hypothesis testing, the test statistic (e.g. *F*, *t*, *r*) with confidence intervals, effect sizes, degrees of freedom and *P* value noted<br>*Give P values as exact values whenever suitable.*
☒	☐	For Bayesian analysis, information on the choice of priors and Markov chain Monte Carlo settings
☒	☐	For hierarchical and complex designs, identification of the appropriate level for tests and full reporting of outcomes
☐	☒	Estimates of effect sizes (e.g. Cohen's *d*, Pearson's *r*), indicating how they were calculated

Our web collection on statistics for biologists contains articles on many of the points above.

Software and code

Policy information about availability of computer code

Data collection	Imaging was acquired using commercially available software based on the GE platform used for these studies.
Data analysis	Data were reconstructed in MATLAB R2018b (The MathWorks, Inc., Natick, MA).<br>Tumor regions of interest (ROIs) were generated using OsiriX 10.0 (Pixmeo SARL, Bernex, Switzerland).<br>HALO v3.2.1851.266 (Indica Labs, Albuquerque, NM, USA) was used for immunohistochemistry and RNAscope analyses (the latter was performed using the FISH v2.2.0 module).<br>Statistical analyses were performed in GraphPad Prism (version 9.0.2, GraphPad Software, San Diego, CA, USA)

For manuscripts utilizing custom algorithms or software that are central to the research but not yet described in published literature, software must be made available to editors and reviewers. We strongly encourage code deposition in a community repository (e.g. GitHub). See the Nature Portfolio guidelines for submitting code & software for further information.

Data

Policy information about availability of data

All manuscripts must include a data availability statement. This statement should provide the following information, where applicable:

- Accession codes, unique identifiers, or web links for publicly available datasets
- A description of any restrictions on data availability
- For clinical datasets or third party data, please ensure that the statement adheres to our policy

The authors declare that the main data supporting the findings of this study are available within the article and its Supplementary Information. The open-source TCGA-PRAD data used in this study are available on cBioPortal [https://www.cbioportal.org/study/summary?id=prad_tcga]. The source data underlying Figures 3, 4, 6, as well as Supplementary Figures 3 and 5 are provided as Source Data.

Field-specific reporting

Please select the one below that is the best fit for your research. If you are not sure, read the appropriate sections before making your selection.

☒ Life sciences ☐ Behavioural & social sciences ☐ Ecological, evolutionary & environmental sciences

For a reference copy of the document with all sections, see nature.com/documents/nr-reporting-summary-flat.pdf

Life sciences study design

All studies must disclose on these points even when the disclosure is negative.

Sample size	For the purpose of this pilot physiological study, with the primary objective of assessing the clinical feasibility of the MRI techniques, insufficient prior knowledge from clinical data was available to perform a formal sample size calculation. The indicative sample size was determined based on the available pre-clinical data and considerations of the anticipated recruitment rates over the specified study duration.
Data exclusions	Two tumours were excluded from the imaging analysis due to technical failure of the HP-13C-MRI. Tissue samples from one patient were excluded from the analysis based on the likely impact of the interval androgen deprivation therapy on the histopathological mechanisms investigated in this study. This exclusion could not be foreseen because the interval androgen deprivation therapy was prescribed due to cancellation of all elective surgeries during the COVID-19 pandemic and was, therefore, not part of a standard of care. Several samples were excluded from single-gland based HIF-1a and PDHA1 analyses due to failure of IHC/RNAscope or insufficient amount of tissue samples left for the post-hoc analyses. These data exclusions are described in the manuscript.
Replication	The hyperpolarized imaging data was acquired following a single injection and could not be repeated. However, images were acquired at multiple timepoints following injection which reduced the effects of noise or artefact in a single image.
Randomization	Randomization was not undertaken as there was no therapeutic intervention. Consecutiive patients fulfillling the required inclusion and exclusion criteria and consenting to be enrolled were included to avoid investigator bias.
Blinding	Investigators who performed IHC and RNAscope analyses were blinded to group allocation during data collection.

Reporting for specific materials, systems and methods

We require information from authors about some types of materials, experimental systems and methods used in many studies. Here, indicate whether each material, system or method listed is relevant to your study. If you are not sure if a list item applies to your research, read the appropriate section before selecting a response.

Materials & experimental systems

n/a	Involved in the study	
☐	☒	Antibodies
☒	☐	Eukaryotic cell lines
☒	☐	Palaeontology and archaeology
☒	☐	Animals and other organisms
☐	☒	Human research participants
☒	☐	Clinical data
☒	☐	Dual use research of concern

Methods

n/a	Involved in the study	
☒	☐	ChIP-seq
☒	☐	Flow cytometry
☒	☐	MRI-based neuroimaging

Antibodies

Antibodies used	MCT1 (Cat. No. HPA003324, Atlas Antibodies, Bromma, Sweden) diluted to 0.2 µg/ml<br>MCT4 (Cat. No. HPA021451, Atlas Antibodies, Bromma, Sweden) diluted to 0.6 µg/ml<br>HIF-1 alpha (Cat. No. ab51608, Abcam, Cambridge, UK) diluted to 23.36 µg/ml<br>Anti-rabbit Poly-HRP-IgG (component of Leica Biosystems Polymer Refine Detection System, DS9800)
Validation	MCT1 antibody has enhanced orthogonal data for IHC, as carried out via the Human Protein Atlas analysis: https://www.proteinatlas.org/ENSG00000155380-SLC16A1/antibody. In-house IHC validation was carried out using a rat brain with a human tumour, with a good cell surface signal observed in the tumour with both sodium citrate and tris EDTA pre-treatment. Additional validation was conducted in a human xenograft in a rat host, with the slides reviewed by a consultant pathologist following the identification of appropriate sodium citrate and tris EDTA concentrations.<br>MCT4 antibody has approved IHC validation data, as carried out via the Human Protein Atlas analysis: https://www.proteinatlas.org/ENSG00000141526-SLC16A3/antibody. In-house validation was carried out in a similar fashion to the MCT1 antibody, with cell membrane signal observed and the slides reviewed by a consultant pathologist.<br>HIF-1 alpha antibody was optimised in collaboration with a pathologist, using breast tissue (both normal and tumour) as controls, along with a TMA derived from the METABRIC database. The positive cases had either nuclear or cytoplasmic staining.

Human research participants

Policy information about studies involving human research participants

Population characteristics	The study included ten consecutive patients with MR-visible (> 1 cm) histologically proven prostate cancer scheduled for radical prostatectomy. All patients enrolled in the study were male, with the median age being 65 years (interquartile range 62.5-67.8 years).
Recruitment	Suitable patients were selected at clinical multi-disciplinary team meetings or clinics and approached by clinical staff involved in their routine care. If patients agreed, they were then approached by the research staff when the nature of the study was explained and they were given written information to read. Patients were given at least 24 hours to consider the study before consenting. Consecutive patients suitable for imaging were consented when the study was open for recruitment. Only patients with MR-visible (> 1 cm) lesions were included in this study, which was based on the spatial resolution acquired using 13C-MRI; this selection bias is unlikely to influence the study results since the proposed clinical use of the technique is only relevant to patients with MR-visible tumours that require additional assessment of their aggressiveness.
Ethics oversight	This prospective study was approved by the institutional review board (National Research Ethics Service Committee East of England, Cambridge South, Research Ethics Committee number 16/EE/0205). The study is managed by an oversight committee and local R&D policies.

Note that full information on the approval of the study protocol must also be provided in the manuscript.
